# Urolithiasis in Kidney Transplant Patients: A Multicenter KSER Research Series

**DOI:** 10.3390/medicina60010132

**Published:** 2024-01-10

**Authors:** Kang Hee Shim, Kwi Bok Choi, Woong Bin Kim, Seung Woo Yang, Do Kyung Kim, Min Soo Choo, Doo Yong Chung, Hae Do Jung, Sin Woo Lee, Bum Soo Kim, Seung Hyun Jeon, Seok Ho Kang, Sunghyun Paick, Joo Yong Lee

**Affiliations:** 1 Department of Urology, Ajou University School of Medicine, Suwon 16499, Republic of Korea; jenius85@hanmail.net; 2 Department of Urology, National Police Hospital, Seoul 05715, Republic of Korea; acala0905@gmail.com; 3 Department of Urology, Soonchunhyang University Bucheon Hospital, Soonchunhyang University College of Medicine, Bucheon 14584, Republic of Korea; woongbins@schmc.ac.kr; 4 Department of Urology, Chungnam National University College of Medicine, Daejeon 35015, Republic of Korea; white0941@hanmail.net; 5 Department of Urology, U-well Urology Clinic, Daejeon 35233, Republic of Korea; 6 Department of Urology, Soonchunhyang University Hospital, Soonchunhyang University College of Medicine, Seoul 04401, Republic of Korea; dokyung@schmc.ac.kr; 7 Department of Urology, Seoul Metropolitan Government, Seoul National University Boramae Medical Center, Seoul 07061, Republic of Korea; mschoo@snu.ac.kr; 8 Department of Urology, Inha University College of Medicine, Incheon 22212, Republic of Korea; dychung@inha.ac.kr; 9 Department of Urology, Inje University Ilsan Paik Hospital, Inje University College of Medicine, Goyang 10380, Republic of Korea; haedojung@paik.ac.kr; 10 Department of Urology, Seoul Medical Center, Seoul 02053, Republic of Korea; icinoo0923@naver.com; 11 Department of Urology, School of Medicine, Kyungpook National University, Daegu 41944, Republic of Korea; urokbs@knu.ac.kr; 12 Department of Urology, Kyung Hee University School of Medicine, Seoul 02447, Republic of Korea; juro@khu.ac.kr; 13 Department of Urology, Korea University College of Medicine, Seoul 02841, Republic of Korea; mdksh@korea.ac.kr; 14 Department of Urology, Konkuk University School of Medicine, Seoul 05030, Republic of Korea; 15 Department of Urology, Severance Hospital, Urological Science Institute, Yonsei University College of Medicine, Seoul 03722, Republic of Korea; 16 Center of Evidence Based Medicine, Institute of Convergence Science, Yonsei University, Seoul 03722, Republic of Korea

**Keywords:** acute renal injury, renal transplantation, urolithiasis

## Abstract

*Background and Objectives*: Urolithiasis occurrence is uncommon in kidney transplantation patients, though it has serious implications, including acute kidney injury in the transplanted kidney. This study investigates the leading causes of urolithiasis in kidney transplantation patients, the diagnostic process, and the outcomes of multimodal management. *Materials and Methods*: Data collection spanned from January 1997 to December 2021, involving kidney transplantation patients with urolithiasis from the database of the Korean Society of Endourology and Robotics (KSER) research committee. Analysis encompassed factors triggering urolithiasis, the diagnostic process, stone attributes, treatment methods, and outcomes. *Results*: Our analysis included 58 kidney transplantation patients with urolithiasis from eight medical centers. Of these patients, 37 were male and 4 had previous urolithiasis diagnoses. The mean age was 59.09 ± 10.70 years, with a mean duration from kidney transplantation to diagnosis of 76.26 ± 183.14 months. The most frequent method of stone detection was through asymptomatic routine check-ups (54.7%). Among the 58 patients, 51 underwent stone treatment. Notably, 95.3% of patients with ureter stones received treatment, a significantly higher rate than the 66.7% of patients with renal stones (*p* = 0.010). Success rates showed no significant differences between renal (70%) and ureter stone (78.0%) groups (*p =* 0.881). *Conclusions*: Urolithiasis in transplanted kidneys constitutes an acute condition requiring emergency intervention. Endo-urological interventions are effective for kidney transplantation patients with urolithiasis. To ensure prevention and early detection, diligent follow-up and routine imaging tests are necessary.

## 1. Introduction

Kidney transplantation (KT) is the most effective treatment for patients with renal failure. The advancement of diverse immunosuppressants in post-transplant management has yielded favorable outcomes, contributing to prolonged graft survival and diminished transplant-related complications [1,2,3,4,5]. Urolithiasis is one of the representative diseases in urology and has a high incidence worldwide, and its incidence and prevalence have been increasing recently. [6] Additionally, urolithiasis ranks among the prevalent conditions capable of inflicting renal damage, and numerous recommendations exist for its diagnosis, treatment, and prevention [7]. However, the applicability of these recommendations to KT patients is somewhat limited due to the unique context of a single kidney. The incidence of urolithiasis in KT patients is reportedly very low, ranging from 0.4% to 2.4%. Nonetheless, it represents a serious disease that can lead to fatal consequences through acute kidney injury in a transplanted kidney.

Several factors have been identified as affecting the occurrence of stones in transplanted kidneys. The direct causes and comorbidities of stone occurrence include foreign body nidus (such as remnant suture materials or ureteral stents), donor-related lithiasis, metabolic diseases, oliguria, recurrent urinary tract infections (UTIs), and chronic urinary tract obstructions [8,9,10]. Most urolithiasis cases in KT patients are typically asymptomatic, unlike general urolithiasis patients who commonly experience pain as the primary symptom. Therefore, the diagnostic process is different between KT and general urolithiasis patients. Moreover, the anatomical structure of the deformed ureterovesical anastomosis site following transplantation presents various challenges during the treatment process.

Advances in technology and the availability of various equipment, including lasers, have significantly transformed the surgical treatment of urolithiasis today [11,12]. Flexible ureteroscopy (fURS) has enhanced access to stones. Additionally, the introduction of mini-percutaneous nephrolithotomy (PCNL) has indicated relatively higher safety levels when compared to standard PCNL. These advancements in treatment methods have opened up possibilities for a more varied and proactive approach to managing urolithiasis in KT patients.

Since the inception of KT in Korea in 1969, the number of kidney transplants has steadily risen. By 2016, the annual count of kidney transplants had exceeded 2000 cases [13]. Consequently, it is anticipated that the overall incidence of urolithiasis in KT patients will increase, despite the relatively low prevalence of kidney stones in transplant recipients. However, there has been a scarcity of studies addressing urolithiasis in KT patients. Therefore, our study aimed to investigate the causes, diagnostic processes, and treatment methods for a large multicenter cohort of KT patients with urolithiasis. Furthermore, we sought to assess the success rate of various treatment methods based on the stone’s location to identify the most effective approach.

## 2. Materials and Methods

### 2.1. Patient Cohort

This study was approved by the Institutional Review Board (approval number: AJIRB-MED-MDB-22-005). We collected datasets for KT patients with urolithiasis from the Korean Society of Endourology and Robotics (KSER) research committee database. The data collection period ranged from January 1997 to December 2021. By using diagnostic codes, we identified patients based on the International Classification of Diseases, Ninth Revision, Clinical Modification (ICD-9-CM). Specifically, we reviewed the records of all patients diagnosed with both Z94.0 (kidney transplant status) and N20-N23 (urolithiasis). Patients with stones in their native kidneys before transplantation were excluded, and only those with confirmed transplanted kidney or ureter stones detected through imaging studies were included.

### 2.2. Classification from Cohort

To ascertain the causes of urolithiasis in transplanted kidneys, we examined patient-related risk factors identified in previous studies [10], categorizing them into direct causes and comorbidities. Direct causes encompass factors such as urolithiasis originating from a cadaveric donor, residual suture material, and prolonged presence of ureteral stents. Comorbidities included oliguria, recurrent UTIs, stenosis, voiding difficulties, and vesicoureteral reflux. Recurrent UTI was defined as a situation in which a patient had been hospitalized three or more times due to UTIs after transplantation. Stenosis was defined as the continuous confirmation of hydronephrosis beyond grade 1 during postoperative follow-up imaging studies. Voiding difficulty was defined by recorded collaboration with the urology department or the ongoing administration of alpha-blockers after KT. In addition, recognizing that the diagnostic process in KT patients differs from that in general patients, we also investigated the time elapsed from transplantation to diagnosis, the rationale behind conducting the examination, and the imaging tools employed to diagnose the stones. The stone component analysis presented the quantitative analysis results of the obtained stone samples in terms of percentage. The stone composition was classified into six main groups according to the Mayo Clinic classification [14].

### 2.3. Treatment for Stone Disease

To determine the optimal treatment based on stone location, we categorized patients into two groups: kidney stones and ureter stones. Patients with stones present in both the kidney and ureter were included in the ureter stones group. We assessed the necessity of emergency intervention, the selection of treatment methods, and their outcomes to discern potential differences between these two groups. Additionally, in identifying factors associated with successful stone treatment, we divided patients who underwent procedures into two categories: the stone-free group and the non-stone-free group. Stone-free was defined by the presence of residual stones with a diameter of no greater than 2 mm along the major axis, as confirmed by CT scans.

### 2.4. Statistical Analysis

For quantitative variables, data are presented as means ± standard deviation. Qualitative variables are presented as percentages. To perform statistical analysis, we employed the chi-square test, Fisher’s exact test, and Mann–Whitney U test using SPSS version 18.0 (IBM, Chicago, IL, USA). Statistical significance was set at *p* < 0.05.

## 3. Results

### 3.1. Demographic Data

We analyzed a total of 58 KT patients with urolithiasis from eight medical centers. Table 1 presents the demographic data for all KT patients with urolithiasis. Among them, 37 patients were male, and 4 patients had a previous urolithiasis diagnosis. The mean age was 59.09 ± 10.70 years, and the mean period from KT to diagnosis was 76.26 ± 183.14 months (Table 1).

### 3.2. Stone Characteristics

The primary mode of stone detection was through asymptomatic routine check-ups, accounting for the highest proportion at 51.7%. Hematuria, pain, and UTI constituted 24.1%, 6.9%, and 5.2% of cases, respectively. Abdominal CT scans were performed in 50 patients, and ultrasound was utilized for stone detection in 7 patients. A single stone was identified in 33 patients, whereas 25 patients had two or more stones. Kidney stones and ureter stones were found in 15 and 35 patients, respectively, and 8 patients had stones present in both the kidney and ureter. At the time of diagnosis, hydronephrosis of greater than grade 1 was observed in 48 patients.

### 3.3. Stone Composition

**Figure 1:**
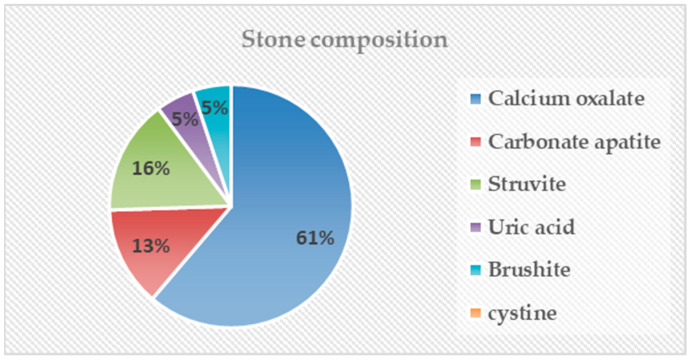
Stone composition by Mayo Clinic classification.

Among 58 patients, analysis was conducted on 21 patients with stone analysis results and presented using Stone composition by Mayo Clinic classification [14]. Pure stone components were confirmed in 11 of 21 patients. Calcium oxalate was confirmed in eight patients, and struvite, uric acid, and brushite were confirmed in one patient each. Calcium oxalate stones were the most common, occurring in 61% of patients, followed by struvite stones and carbonate apatite stones at 16% and 15%, respectively. Uric acid stones and brushite stones each occurred in 5% of patients, and cystine stones were not identified (Figure 1).

### 3.4. Causes of Stone Formation

In this study, among the direct causes of stone formation, ureteral stents left in place for an extended period were confirmed in four cases, while urolithiasis originating from a cadaveric donor was identified in two cases. Notably, there were no cases of urolithiasis resulting from remnant suture materials. In terms of comorbidities associated with urolithiasis, there was one case of oliguria, one case of recurrent UTI, two cases of stenosis, and two cases of voiding difficulty (Table 2).

### 3.5. Management of Disease

Emergency intervention was required for 25 patients, with 19 patients undergoing percutaneous nephrostomy insertion, while the remaining 6 patients received ureteral stent insertion. Out of the total 58 patients, 51 underwent stone treatment, which included the utilization of extracorporeal shockwave lithotripsy (ESWL). As the initial treatment approach, 23 patients were treated with ureteroscopic surgery, 14 with percutaneous PCNL, 12 with ESWL, and 2 with alternative methods.

### 3.6. Treatment Outcome

To assess the effectiveness of the initial treatment choice and its success rate based on stone location, the 51 treated patients were categorized into two groups: the renal stone group (15 patients) and the ureter stone group (42 patients). Analysis of their outcomes is presented in Table 3. In the ureter stone group, 95.3% of patients received stone treatment, which was significantly higher than in the renal stone group, in which 66.7% of patients were treated (*p* = 0.010). The necessity for emergency intervention was significantly higher in the ureter stones group at 51.2% compared to the 20% observed in the kidney stones group (*p* = 0.036). However, there was no significant difference in the choice of treatment based on stone location (*p* = 0.562). Similarly, there was no significant difference between the two groups in terms of the stone-free rate (*p* = 0.924) (Table 3). Furthermore, when comparing non-stone-free and stone-free patients, there were significant statistical differences between the two groups in terms of target stone volume and mean stone length (*p* = 0.004 and *p* = 0.003, respectively). However, there were no differences between the two groups in terms of mean age, body mass index, detection period after KT, and mean stone density. (Table 4).

## 4. Discussion

Our study delved into the direct causes and comorbidities, the diagnostic process, the need for emergency intervention, and treatment modalities and their success rates in 58 KT patients with urolithiasis. Additionally, we analyzed the practical treatments and their success rates based on the stone’s location, identifying factors affecting the success of the stone treatment. Our investigation contributes valuable insights to the limited but significant body of research regarding the diagnostic and treatment strategies for urolithiasis in KT patients. A comparison of previous studies with ours is presented in Table 5.

Contrary to initial expectations, we did not observe a statistically significant difference in the selection of the primary treatment methods and their success rates based on stone location. Furthermore, the treatment success rate was relatively low compared to those in previous studies [1,3]. This finding necessitates further consideration. It is worth noting that the success rate of the treatments assessed in our study reflects outcomes after only the initial treatment session. Additionally, due to the relatively small total patient population in our study, the results obtained may be less conclusive due to statistical limitations. However, the absence of variations in treatment choice and success rates by stone location suggests that the treatments selected in this study may indeed represent the most suitable modalities. Successful stone treatment depends on multiple factors, including stone location, stone size, its relationship with surrounding structures, and the patient’s overall condition. Further research in these areas is expected to yield improved results.

The causes of stones and comorbidities in KT patients differ somewhat from those in non-KT patients. Common causes for stone occurrence in transplanted kidneys include receiving a kidney transplant with pre-existing stones or experiencing complications such as infection and obstruction after KT [15,16]. In 1985, Van Gansbeke et al. [15] first introduced the concept of donor-graft lithiasis and emphasized the importance of careful follow-up to preserve renal function in cases of kidney stones. Torrecilla Ortiz et al. [17] suggested that even if stones were found in a cadaveric donor kidney, this should not be a reason to refuse the graft, as it could be effectively treated through appropriate endo-urological interventions. More recently, Henderickx et al. [18] reported successful kidney stone removal by performing back-table endoscopy in a renal allograft. In our study, two stones originating from the donor kidney were effectively treated using ESWL and PCNL, respectively.

In 2012, Verrier et al. [16] analyzed surgical risk factors directly influencing stone occurrence. Their study revealed a significant decrease in the incidence of kidney stones from 2.1% to 0.6% over three decades. They attributed this reduction to the preventive use of perioperative ureteral stents and early treatment of ureteral obstruction. However, in our present study, four cases of stones were attributed to prolonged ureteral stent placement, highlighting the need for even more vigilant follow-up despite the potential positive impact of perioperative ureteral stent insertion in reducing obstructions.

The clinical presentations of urinary stones in KT patients exhibit notable differences compared to those in non-KT patients. Challacombe et al. [1] reported that urolithiasis in KT patients often manifests with atypical symptoms, often without the typical symptom of pain. However, with increased awareness of this condition, early-stage diagnosis has become possible. In a study conducted by Emiliani et al. [19], typical clinical symptoms of urolithiasis such as pain and hematuria were observed in only 4% and 23% of cases, respectively, while the highest rate (43%) involved incidental diagnosis during follow-up. These findings closely resemble the clinical features identified in our study: asymptomatic cases detected during routine check-ups (55%), hematuria (24%), and pain (5%). Furthermore, the cause of detection was categorized based on the presence or absence of symptoms, and an analysis was conducted to determine if there were differences in the time until diagnosis and the size of the stones found. However, no statistically significant differences were observed between the two groups (*p* = 0.527 and *p* = 0.354, respectively). Nevertheless, one crucial point remains evident: diligent clinical and imaging assessments are imperative for the early detection and treatment of urinary stones in KT patients.

**Table 5:** Summary of previous studies on urolithiasis in kidney transplant patients.

Author	StudyDesign	Country	Duration	Stone Patients (n)	Prevalence (%)	Mean Age (Years)	Stone Location (*n*)	Mean Stone Size	Any Procedure (n)	Treatment Modality (n)	SFR (%)
Yuan et al. [3]	Single center	China	2000–2014	19	1.20%	38	Kidney 9 (47.34%)Ureter 9 (47.34%)Both 1 (5.2%)	4.2 mm	17/19 (89.5%)	SWL 5 (29.4%)URS 5 (29.4%)PCNL 7 (41.2%)	100
Challacombe et al. [1]	Single center	UK	1977–2003	21	1%	41	N/A	8.1 mm	19/21 (90.5%)	SWL 12 (63.2%)URS 2 (10.5%)PCNL 3 (15.8%)Others 2 (10.5%)	100
Emiliani et al. [19]	Single center	Spain	1983–2017	51	2.40%	48.9	Kidney 19 (37.25%)Ureter 32 (62.75%)	9 mm	37/51 (54.4%)	SWL 22 (59.5%)URS 9 (24.3%)PCNL 4 (10.8%)others 2 (5.4%)	52.9
Verrier et al. [15]	Single center	France	1978–2010	31	1.03%	40.5	Kidney 11 (35.5%)Ureter 16 (51.6%)Bladder 4 (12.9%)	N/A	19/31 (61.3%)	SWL 3 (15.8%)URS 5 (26.3%)PCNL 1 (5.2%)Others 10 (51.7%)	58.1
Current study	Multi center	Korea	1997–2021	58	N/A	59.1	Kidney 15 (25.9%)Ureter 35 (60.3%)Both 8 (13.8%)	575.8 mm³	51/58 (87.9%)	SWL 12 (23.5%)URS 23 (45.1%)PCNL 14 (27.5%)Others 2 (3.9%)	60.8

SWL: shockwave lithotripsy, URS: ureteroscopic surgery, PCNL: percutaneous nephrolithotomy.

Recently, there was a study in Korea that analyzed stone composition in a large group of patients [20]. According to the results of this study, calcium oxalate (46%) had the highest proportion, followed by struvite (29%) and uric acid (19%). Carbonate apatite, brushite, and cystine comprised less than 5% of the total. This is somewhat different from the composition of stones identified in our study. The results of the high proportions of calcium oxalate and struvite are similar, but the difference is that the proportion of uric acid stones is low at 5% and the proportion of carbonic apatite is high at 15%. There are several assumptions that can lead to this result. First, struvite and carbonic apatite are representative stones known to be caused by infectious microorganisms [21]. In fact, in this study, three out of four patients with carbonated apatite that was confirmed to be in a mixed form with struvite. In renal transplant patients, the immunosuppressed state due to the use of immunosuppressants may have caused stones due to repeated urinary tract infections. Second, the recommendation to consume a large amount of water to maintain the function of the transplanted kidney [22] may have reduced the occurrence of uric acid stones, which are relatively greatly affected by water intake [23]. In fact, the stone composition of transplant kidneys was different from that of autologous kidney patients, suggesting that the causes of stone formation in transplant patients may be different. It will be necessary to find metabolic factors that cause stones in transplanted kidneys through additional research.

While the treatment for common kidney stones in transplanted kidneys may share some similarities with that of single kidneys, there are distinct differences that must be taken into account. First, there is a disparity in endoscopic accessibility due to structural changes at the ureterovesical anastomosis site. Accessing the transplanted kidney can be challenging due to the presence of a deformed neo-ureteric orifice and the pelvic positioning of the transplanted kidney. The development of endoscopic technology and the introduction of fURS have improved retrograde access to the neo-ureteral orifice, achieving a stone-free rate (SFR) of close to 90% [19,24]. In our study, the SFR of fURS was relatively lower at 52.2%, but factors influencing the SFR, such as stone location, size, and composition, were not taken into account. The strict SFR criteria in this study, which required the size of the residual stone to be less than 2 mm, may have contributed to the lower SFR.

Second, the reduction in skin-to-stone distance (SSD) due to the transplanted kidney’s superficial positioning, as opposed to its original location, is another crucial consideration when treating kidney stones in transplanted kidneys. ESWL is one of the least invasive treatment modalities and can be highly effective, with success rates ranging from 87% to 100% when the stone’s size and location are appropriately assessed [8,25]. Patel et al. [26] indicated that SSD is an independent predictor of stone-free status following ESWL, with statistical significance observed in the SFR between groups with mean SSDs of 83.3 ± 21.9 mm and 107.7 ± 28.9 mm. In our study, the mean SSD was 57.99 ± 17.16 mm, which is considered favorable for ESWL treatment. Additionally, the success rate reached 67.7%, which is relatively high, especially considering that the SFR was determined after the initial ESWL treatment session only.

The possibility of direct access to stones, excluding deformed ureters, and enhanced accessibility due to the relatively reduced SSD indicate that PCNL can be a viable treatment modality for KT patients. However, despite its excellent advantages, such as the potential for complete removal of the stone burden, the selection of PCNL is not straightforward due to the potential risk of damaging the single kidney. The emergence of minimally invasive PCNL (mPCNL), which demonstrated significantly lower risks compared to traditional PCNL [27], has made it a safer and more efficient option for treating urolithiasis in transplanted kidneys [28]. In addition, ureteroscopy-assisted puncture for ultrasonography-guided renal access has further improved safety and overall treatment outcomes [29]. In our study, PCNL, including both mPCNL and endoscopic combined intrarenal surgery, was selected as the first-line treatment for 14 patients, representing a significant portion of the treatment choices compared to ESWL.

Our study has several limitations, including its retrospective design and a patient cohort that remains relatively small, despite being large compared to similar studies. Given the inherently low prevalence of this condition, conducting a prospective study on stone disease in KT patients in the future is expected to be challenging. Furthermore, the absence of prevalence as one of the major etiologies represents a limitation of our study. Therefore, additional supplementation through alternative research methods, such as meta-analysis, is necessary. Additionally, one of the important limitations of this study is the lack of results for metabolic studies such as 24-h urine collection. These are essential data in the process of studying the etiology of stone occurrence, but the results were not presented in this study due to the limitations of the retrospective study. As we reviewed and analyzed medical records written a long time ago, we had no choice but to conduct evaluations based on record sheets and images rather than test results. For the same reason, outcome analysis was also conducted with a greater focus on diagnosis and treatment of the stones.

Our study stands out in its unique approach, wherein primary treatments were practically administered by various physicians to 58 KT patients across multiple centers. We then analyzed the success rates of these treatments based on the stone’s location in KT patients. Furthermore, by analyzing whether PCN or ureteral stent insertion was required, we confirmed that over half of the ureteral stones in KT patients presented as acute diseases requiring emergency intervention. Comparatively, endo-urological interventional treatments have proven effective in KT patients with urolithiasis when compared to non-KT patients. Our study contributes valuable evidence for the diagnosis of urolithiasis and the development of treatment strategies for various scenarios in KT patients.

## 5. Conclusions

Urolithiasis in transplanted kidneys represents an acute condition that necessitates emergency intervention. Furthermore, endo-urological interventional treatments have proven to be effective in KT patients with urolithiasis. We anticipate that our study will provide valuable insights for treatment decision-making. For prevention and early detection of urolithiasis in KT patients, it is imperative to maintain diligent follow-up schedules and conduct regular imaging tests.

## Figures and Tables

**Table 1:** Demographic data of kidney transplant patients with urolithiasis.

	Total
Number of patients	58 (100.0%)
Sex	
Male	37 (63.8%)
Female	21 (36.2%)
Previous stone history	4/58 (6.9%)
Age (years)	59.09 ± 10.70
BMI (kg/m^2^)	23.37 ± 3.37
Detection period after KT (months)	76.26 ± 183.14
Mode of stone detection	
Asymptomatic routine check-up	30 (54.7%)
Hematuria	14 (24.1%)
Pain	4 (6.9%)
Urinary tract infection	3 (5.2%)
Other	7 (12.1%)
Multiplicity	
Single	33 (56.9%)
Multiple	25 (43.1%)
Location	
Kidney	15 (25.9%)
Ureter	35 (60.3%)
Both	8 (13.8%)
Hydronephrosis	48 (82.8%)
Volume of target stone (mm^3^)	575.84 ± 1739.69
MSL (mm)	11.63 ± 9.83
MSD (HU)	693.40 ± 354.20
Emergency intervention	25 (43.1%)
PCN insertion	19/25 (76.0%)
Ureteral stent insertion	6/25 (24.0%)
Any other procedure	51 (87.9%)
Procedure for initial treatment	
SWL	12 (23.5%)
URS	23 (45.1%)
PCNL	14 (27.5%)
Other	2 (3.9%)

BMI: body mass index, KT: kidney transplantation, MSL: maximal stone length, MSD: mean stone density, HU: Hounsfield units, PCN: percutaneous nephrostomy, SWL: shockwave lithotripsy, URS: ureteroscopic surgery, PCNL: percutaneous nephrolithotomy.

**Table 2:** Direct causes and comorbidities of urolithiasis in kidney transplant patients.

	Number of Patients
Direct causes	
Ureteral stent (left for a prolonged period)	4 (6.8%)
Cadaveric donor	2 (3.4%)
Remnant suture material	0 (0.0%)
Comorbidity	
Oliguria	1 (1.7%)
Recurrent urinary tract infection	1 (1.7%)
Stenosis	2 (3.4%)
Voiding difficulty	2 (3.4%)
Vesicoureteral reflux	0 (0.0%)

**Table 3:** Comparison of treatment method, initial treatment option choices, and treatment outcomes according to the stone location in kidney transplant patients.

	Renal Stone Group	Ureter Stone Group	*p*-Value
Total number of patients	15 (25.9%)	43 (74.1%)	
Treatment			0.010 ^a^
Conservative care	5 (33.3%)	2 (4.7%)	
Any other procedure	10 (66.7%)	41 (95.3%)	
Need for emergency intervention			0.036 ^a^
No	12 (80.0%)	21 (48.8%)	
Yes	3 (20.0%)	22 (51.2%)	
Initial treatment option			0.562 ^a^
SWL	3 (30.0%)	9 (21.9%)	0.682 ^b^
URS	3 (30.0%)	20 (48.8%)	0.480 ^b^
PCNL	4 (40.0%)	10 (24.4%)	0.432 ^b^
Others	0 (0.0%)	2 (4.9%)	-
Stone-free rate	6/10 (60.0%)	26/41 (63.4%)	0.924 ^a^
Operation success rate	7/10 (70.0%)	32/41 (78.0%)	0.881 ^a^

SWL: shockwave lithotripsy, URS: ureteroscopic surgery, PCNL: percutaneous nephrolithotomy. ^a^ The chi-square test. ^b^ Fisher’s exact test.

**Table 4:** Comparison of non-stone-free and stone-free groups in kidney transplant patients.

Variable	Total	Non-Stone-Free Group	Stone-Free Group	*p*-Value
Number of patients	49	17	32	
Age (years)	58.57 ± 11.01	55.88 ± 11.94	61.00 ± 9.73	0.105
BMI (kg/m^2^)	23.55 ± 3.49	23.30 ± 3.86	23.1 ± 2.47	0.431
Detection period (months)	71.00 ± 191.88	111.35 ± 325.17	49.44 ± 58.00	0.406
Target stone volume (mm^3^)	647.82 ± 1845.49	1311.94 ± 3087.56	298.98 ± 447.27	0.004
MSL (mm)	11.81 ± 10.27	14.34 ± 15.65	10.55 ± 6.18	0.003
MSD (HU)	738.84 ± 344.14	864.91 ± 394.40	680.15 ± 311.74	0.106

Values are presented as numbers only or mean ± standard deviation. BMI: body mass index, MSL: maximal stone length, MSD: mean stone density.

## Data Availability

Data are available upon request to the corresponding author.
